# Nutrition Transition in Europe: East-West Dimensions in the Last 30 Years—A Narrative Review

**DOI:** 10.3389/fnut.2022.919112

**Published:** 2022-07-07

**Authors:** Klara G. Dokova, Rouzha Z. Pancheva, Natalya V. Usheva, Galina A. Haralanova, Silviya P. Nikolova, Todorka I. Kostadinova, Caue Egea Rodrigues, Jessica Singh, Anne-Kathrin Illner, Krasimira Aleksandrova

**Affiliations:** ^1^Department of Social Medicine and Health Care Organization, Faculty of Public Health, Medical University Prof. Dr P. Stoyanov, Varna, Bulgaria; ^2^Department of Hygiene and Epidemiology, Faculty of Public Health, Medical University Prof. Dr P. Stoyanov, Varna, Bulgaria; ^3^Department of Economics and Health Care Management, Faculty of Public Health, Medical University Prof. Dr P. Stoyanov, Varna, Bulgaria; ^4^Department of Pharmacology and Toxicology, Institute of Pharmacy, Freie Universität Berlin, Berlin, Germany; ^5^Cancer Research and Clinical Trials, Ballarat Health Services, Ballarat, VIC, Australia; ^6^Department of Sport, Exercise and Nutrition Sciences, School of Allied Health, Human Services and Sport, La Trobe University, Bundoora, VIC, Australia; ^7^College of Health Sciences, UniLaSalle, Beauvais, France; ^8^Department Epidemiological Methods and Etiological Research, Leibniz Institute for Prevention Research and Epidemiology -BIPS, Bremen, Germany; ^9^Faculty of Human and Health Sciences, University of Bremen, Bremen, Germany

**Keywords:** nutrition transition, Europe, East-West gaps, dietary changes, review

## Abstract

The current review aims to summarize published research on nutrition transition patterns (depicting changes in dietary consumption) in European populations over the last three decades (1990–2020), with a focus on East-West regional comparisons. Pubmed and Google-Scholar databases were searched for articles providing information on repeated dietary intakes in populations living in countries across Europe, published between January 1990 and July 2021. From the identified 18,031 articles, 62 were found eligible for review (17 from Eastern and 45 from Western European populations). Overall, both in Eastern and Western Europe, there have been pronounced changes in dietary consumption patterns over the last three decades characterized by reductions in average reported intakes of sugar, carbohydrates and saturated fats and increases in reported fruit and vegetable consumption. There has also been a tendency toward a reduction in traditional foods, such as fish, observed in some Mediterranean countries. Overall, these data suggests that European countries have undergone a nutrition transition toward adopting healthier dietary behaviors. These processes occurred already in the period 1990–2000 in many Western European, and in the last decades have been also spreading throughout Eastern European countries. Firm conclusions are hampered by the lack of standardized methodologies depicting changes in dietary intakes over time and the limited coverage of the full variety of European populations. Future studies based on standardized dietary assessment methods and representative for the whole range of populations across Europe are warranted to allow monitoring trends in nutrition transition within and among European countries.

## Introduction

The recent decades have been characterized by dramatic changes in the economic and social outlook of Europe (1). Increasing health inequalities both within and between countries continue to be a challenge and despite decreasing overall national mortality rates, a clear East-West gradient in the population health remains to exist (2). An example is the life expectancy gap between East and West European countries which is even greater for men than it was four decades ago (3, 4). Additionally, the study of Mackenbach et al. (2) found that the East-West difference in morbidity and mortality is extremely high among lowly educated, middle-aged population groups.

Environmental, political, social, educational and economic circumstances have all played a major role in the epidemiological transition (5). Dietary changes resulting from the introduction of a market economy in Eastern countries, accompanied by changes in the availability of various food items have been hypothesized as an important candidate explaining existing health disparities between East and West Europe (6–8). Aging, urbanization and migration have posed additional challenge to public health systems across Europe and have contributed to the dynamics in the nutrition and health patterns in the recent decades (9–11).

Previously, Grigg (12) published a study on European regional variations in food consumption noting a trend of convergence of the nutritional composition of diet between Western and Eastern Europe in the decades after the Second World War. However, no recent evaluation of the existing evidence outlining trends in nutrition changes in European populations have been performed. Several studies provided an evaluation of dietary intake in European populations, such as the DAFNE project, based on household budget surveys data, however these studies were based only on a selected list of European countries and did not cover the whole period included in our review (13, 14). The Global Burden of Disease (GBD) study represents another source of valuable information of dietary intakes in the period 1990–2010 for the majority of European countries (15, 16).

To depict dynamics linking nutritional patterns with the socio-demographic and epidemiological shifts, the concept of “nutrition transition” has emerged as a theoretical framework outlining nutrition changes over time, between and within populations (17, 18).

While the nutrition transition phenomenon was extensively explored in various countries across the globe, e.g., Asia, Africa, the Middle East, and Latin America (19–21), there has been little research outlining nutrition transition patterns in Europe. It remains particularly unclear whether specific nutrition transition dynamics can be traced across populations in Eastern and Western Europe over the recent decades and whether socio-economic transitions have influenced these changes. Such research would be especially valuable in guiding prevention strategies and public health policies that address the health inequalities across various populations in Europe, in particular with regard to nutrition-related chronic diseases.

To address these gaps, we aimed to collect and summarize published research on nutrition transition patterns in European populations over the last three decades (1990–2020) with a focus on East-West regional comparisons.

## Methods

The present narrative review is based on the general framework of narrative reviews proposed by Ferrari (22) and complies with the requirements of the Scale for the Assessment of Narrative Review Articles (SANRA) (23).

### Search Strategy

A literature search of the PubMed and Google-Scholar databases (January 1990 to July 2021) was conducted using the following combination of terms and text words [“nutrition(al) transition” OR “nutrition, dietary, (national) survey” OR “dietary change/trend/pattern” OR “dietary behavior”]. Each of the above 1 to 4 key words were combined with the words: Europe, United Kingdom, and the names of each of the 27 European Union member states, i.e., Austria, Belgium, Bulgaria, Croatia, Republic of Cyprus, Czech Republic, Denmark, Estonia, Finland, France, Germany, Greece, Hungary, Ireland, Italy, Latvia, Lithuania, Luxembourg, Malta, Netherlands, Poland, Portugal, Romania, Slovakia, Slovenia, Spain, and Sweden. Additionally, article bibliographies and gray literature sources were searched for relevant publications. Publications accessible in full text were selected for the review.

### Eligibility Criteria

Studies were included based on the following criteria: (1) studies providing information on dietary intakes over time (from repeated measurements in the same sample, or consecutive samples from the same population); (2) studies reporting on populations living in any one of the 27 countries, members of the European Union by the date of last literature search (July 2021) and the UK, as it was a member of the EU for the 29 of the 30 years period; (3) studies reporting on adult populations, i.e., 18 years and older.

The exclusion criteria were: (1) studies focused on specific population subgroups based on age, gender, ethnic group etc. such as children, adolescents, pregnant women, elderly; (2) studies reporting only behavioral data (i.e., knowledge, attitudes, and beliefs studies with no dietary assessment data reported); (3) food safety research studies; (4) studies providing only data on physical activity or body composition with no reference to diet; (5) studies reporting on factors affecting dietary intake and habits and not actual intake; (6) studies on associations between diet and various health outcomes without data on dietary intakes; (7) studies reporting on specific or clinical nutrition models and narrow aspects of diet, such as specific food groups or nutrients e.g., fats, vitamins, etc. and not whole of diet; (8) studies reporting on consumption in specific circumstances only, such as diet consumed away from home; (9) economic research studies (food sales, costs and purchase and advertisements of foods); (10) dietary/nutritional intervention or methodological studies.

### Study Selection

**Figure 1:**
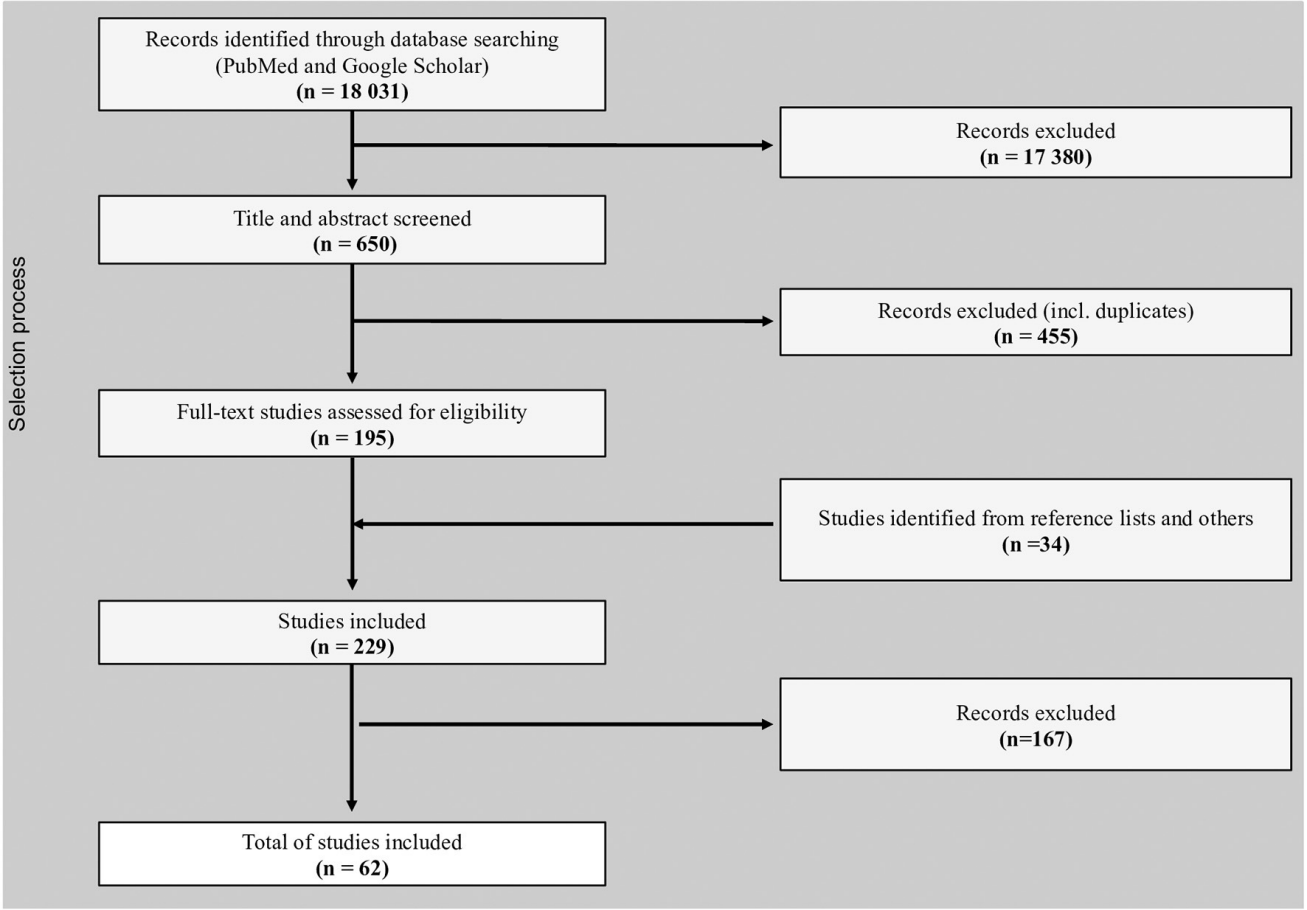
Flow diagram of the stages of the selection process indicating the number of publications at each stage.

The process of study selection, including identification, screening, eligibility, and inclusion is illustrated with a flowchart presented in Figure 1. The search strategy retrieved 18,031 unique records. Based on the initial evaluation, 195 records were selected for full-text detailed evaluation. After manual search of the references of the selected papers and an additional targeted search for publications from the countries for which initially there were no publications fulfilling the criteria, 34 additional articles were identified resulting in 229 articles eligible for full text review. Any discrepancies and disagreements were discussed and resolved by consensus among reviewers. A total of 62 articles were selected for data extraction in the present review.

### Data Extraction

Data extraction was performed by four reviewers (KD, RP, NU, GH) using a predefined data extraction table. The following information was extracted: (1) Country; (2) First author and year of publication; (3) Study design; (4) Time period of the study; (5) Level of data; (6) Number of participants; (7) Age range; (8) Method of dietary assessment. Any discrepancies in the data extraction were discussed and resolved by consensus among the reviewers.

The following information depicting nutrition transition dynamics in the respective European region and individual countries was specifically extracted denoting intakes of specific micro- and macronutrients, and food groups including: (1) “carbohydrates,” incl. bread, bakery products, cereals, rice, pasta, flour, potatoes, potato products; (2) “saturated fatty acids”, incl. animal lipids, butter, added lipids (if no other data); (3) “sugars,” incl. sugar, sugar products, honey, chocolate, and other confectionary”; (4) “fruits and vegetables”; and (5) “fish,” incl. fresh, frozen, canned, fish products. Furthermore, data was extracted according to predefined time periods (decades): (1) 1990–1999; (2) 2000–2009; (3) 2010–2019, and also extracted based on country (categorized into each of the 28 European Union countries). Eastern and Central European countries (referred shortly as Eastern European countries) included those that joined the EU after 2004, characterized by political and economic transition (shifts from non-market to market economies) in the last 30 years. These include the following 11 countries: Bulgaria, Croatia, Czech Republic, Estonia, Hungary, Latvia, Lithuania, Poland, Slovakia, Slovenia and Romania. Only Eastern European EU member states were included in the search due to the recognition that in these countries efforts were concentrated on direct measures to harmonize requirements, including nutritional, and, later, on the establishment of an “internal market.” Subsequently, a concerted shift in emphasis necessitated a greater emphasis on food safety harmonization. Hence, the legislation in Eastern European EU countries was adapted to the EU regulations and the choice for stratification was based on the attempt to standardize the group of participants. The remaining 17 countries included Western European countries traditionally characterized by market economies and representing the initial EU member states.

### Data Evaluation

The identified studies were evaluated according to the theoretical framework proposed by Popkin (17, 24) categorizing the nutrition transition according to 5 patterns: (1) “collecting food pattern” with diets high in carbohydrates and fiber and low in saturated fat; (2) “famine pattern” with diets dominated by cereals and emergence of nutritional deficiencies; (3) “receding famine pattern” which is characterized by low-fat, high-fiber diet with an increase in the consumption of fruits, vegetables, animal protein and a decrease in starchy staples; (4) “degenerative diseases pattern”—diets high in fat, sugar, refined carbohydrates, processed foods, and reduced fiber (5) “behavioral change pattern” characterized with intentional reduction in calorie and fat intake and increased consumption of fruits, vegetables, unrefined carbohydrates accompanied with a higher level of physical activity.

## Results

### Study Characteristics

The final literature search resulted in identification of 62 articles depicting dietary changes in 26 European countries. The main characteristics of the selected studies: name of country and first author, year of publication, time period of study, number of participants, age-range of participants, and method of dietary assessment, are presented according to East vs. West European region in Table 1 (Section A and B). Among all European countries, only Croatia and Luxemburg were not included in the review, due to lack of eligible publications.

**Table 1:** Summary of studies included in the review.

**Section A: Eastern European Countries**					
**Country**	**First author, year (Reference)**	**Time period of study**	**Number of participants**	**Age range, years**	**Method of dietary assessment**
Bulgaria	Ivanova et al. (25)	1985–2002	3,000 to 6,000 hhs	20+	Diary
Bulgaria	Duleva et al. (26)	1998–2014	2,970 in 1998	19+	24-h dietary recall and FFQ
Czeck Republic	Dofkova et al. (27)	1991–1997	420 hhs	N.a.	Diary
Czeck Republic	Ratinger et al. (28)	1950–2012	N.a.	N.a.	N.a
Estonia	Pitsi et al. (29)	2003–2007	3,200 to 3,700 hhs	18+	Interviewer-administered questionnaire and Diary
Hungary	Rodler and Zajkás (30)	1960–1999	2,559	18+	FBS and 24-h dietary recall
Hungary	Szeitz-Szabó et al. (31)	1985–2009	6,815	18+	24-h dietary recall, FFQ
Latvia	Popluga and Melece (32)	1990–2006	N.a.	N.a.	N.a.
Lithuania	Ciapaite et al. (33)	2003–2007	7,000 to 8,000	19+	Diary
Poland	Waśkiewicz et al. (34)	1984–2001	2,571–1,984; 1,397–1,988 1485–1,993; 836–2,001	35–64	Questionnaire
Poland	Jaros et al. (35)	1960–2008	N.a.	N.a.	FBS
Poland	Waśkiewicz et al. (36)	1984–2012	3,404 men; 3,446 women	35–64	24-h dietary recall
Romania	Petrovici and Ritson (37)	1990–1998	36,000 hhs	N.a.	Diary
Slovak Republic	Krizova and Buday (38)	2009–2012	N.a.	N.a.	FBS and HBS
Slovak Republic	Sitarova (39)	2005–2009	N.a.	N.a.	FBS
Slovak Republic	Sitarova (40)	2014–2018	N.a.	N.a.	FBS
Slovenia	Gregoric et al. (41)	1998–2002	1,300 to 3,687 hhs	19+	Diary
**Section B: Western European Countries**					
Austira	Elmadfa (42)	1994–2001	2,580	19–60	24-h dietary recall
Austira	Elmadfa (43)	2003–2008	2,123	18–65	24-h dietary recall
Austira	Elmadfa et al. (44)	2007	2,123	19–64	24-h dietary recall
Belgium	Trichopoulou et al. (45)	1988–1999	N.a.	N.a.	N.a.
Belgium	Temme et al. (46)	1980/5–2004	3,245	15+	24-h dietary recall and FFQ
Belgium	Bel et al. (47)	2004–2014/5	1,226 in 2014/5;	15–64	24-h dietary recall and FFQ
Belgium	Desbouys et al. (48)	2004-2014	1,186 in 2004 952 in 2014	15–39	24-h dietary recall
Cyprus	Markidou et al. (49)	1997–2003	3,308 hhs in 1996/7; 3,599 hhs in 2003	19+	Diary
Denmark	Osler et al. (50)	1982/4–1992/4	3,785 and 7,316	30–70	FFQ
Denmark	Elmadfa et al. (44)	2000–2002	2,769	19–64	Diary 7-day
Denmark	Groth et al. (51)	1995–2008	7,900	20–75	Diary 7-day
Finland	Viinisalo et al. (52)	1966–2006	N.a.	25+	Not specified
Finland	Mannisto et al. (53)	1966–2007	1,861 in 1992 2,862 in 1997 2,007 in 2002 2,039 in 2007	25-64	FBS HBS 3-day record and 24 h recall
France	Perrin et al. (54)	1985/7–1995/7	862 in 1985/7 802 in 1995/7	35-64	3-day record and FFQ
France	Dubuisson et al. (55)	1998/9–2006/7	3,267	18-79	Diary 7-day
Germany	Winkler et al. (56)	1987/8–1991/2	537 and 605	25-64	3-day weighed record/ opened record
Germany	Gedrich et al. (57)	1988–1993–1998	50,000 hhs in 1988, 70,000 in 1993; 62,000 in 1998	19+	Diary of type and quantity of acquired food
Germany	Mensink and Beitz (58)	1991–1998	7,466 in 1991 4,556 in 1998	25–69	FFQ
Germany	Gose et al. (59)	2005/7–2012/3	1,840	14–80	24-h recalls
Greece	Trichopoulou et al. (45)	1988–1999	N.a.	N.a.	N.a.
Greece	Bountziouka et al. (60)	1987/8–1998/9–2004/5	6,555 hhs in 2004/5	19+	Diary of food expenditures and purchases
Greece	Elmadfa et al. (44)	1994–1999	20,399	19–64	FFQ
Greece	Skourlis et al. (61)	1997–2011	23,505	25–86	FFQ, Follow up-dietary questionnaire (FU-DQ)
Ireland	Trichopoulou et al. (45)	1987–1999	N.a.	N.a.	N.a.
Ireland	Sheehy and Sharma (62)	1961–2007	N.a.	N.a.	FBS
Ireland	Flynn et al. (63)	2001–2008/10	1,500	18–90	Diary 4-day semi-weighed
Italy	Trichopoulou et al. (45)	1990–1996	N.a.	N.a.	N.a.
Italy	Elmadfa et al. (44)	n.a.	1,461	19–64	Diary 7-day
Italy	Pelucchi et al. (64)	1991–2006	3,247	18–80	FFQ
Italy	Sette et al. (65)	2005–2006	2,312	18–65	Diary 3-day
Italy	Leone et al. (66)	2010–2016	8,584	18+	FFQ
Italy	Pounis et al. (67)	2010–2013	8,944	18+	24-h recall
Malta	Pace et al. (68)	1994–2000	2,715 Hhs in 1994, 2,748 in 1995, and 2,586 in 2000;	N.a.	Questionnaire and a diary on 3-weeks consumption
Malta	Cauchi et al. (69)	1961–2010	N.a.	N.a.	N.a.
Netherlands	Seidell (70)	1987/8–1997/8	6,218 in 1992; 5,958 in 1997/8	1–100	N.a.
Netherlands	van Rossum et al. (71)	2003–2007/8	2,997	19–70	24-h recalls
Netherlands	van Rossum et al. (72)	2007/10–2012/6	2,395	16–70	24-h recalls
Netherlands	Dinnissen et al. (73)	2007/10–2012/6	2,106; 1,540	19–69	24-h recalls
Portugal	Rodrigues et al. (74)	1990–2000 2000–2005	12,403 hhs 1989, 10,554 in 1994/5 10,020 in 2000/1 10,403 in 2005/6	0–100	Diary
Portugal	Marques-Vidal et al. (75)	1987–1999	39,964 in 1987; 48,873 in 1995/6; 47,179 in1998/9	0–100	24-h recall
Portugal	Bento et al. (76)	1974–2011	N.a.	N.a.	FBS
Spain	Moreno et al. (77)	1964–1991	20,800–28,000 hhs	N.a.	7 day record
Spain	Varela-Moreiras et al. (78)	2000–2006	6,000–8,200 hhs	N.a.	Diary of product acquisition 7 days
Spain	Varela-Moreiras et al. (79)	2000–2012	N.a.	N.a.	Diary of product acquisition 7 days
Spain	Partearroyo et al. (80)	2000–2008	1,587	18–64	Diary 3-day
Sweden	Poortvliet et al. (81)	1989–1996/7	2,970	18–74	Diary of purchased foods amounts for 4 weeks
Sweden	Juul and Hemmingsson (82)	1960–2010	N.a.	N.a.	N.a.
UK	Trichopoulou et al. (45)	1989–1999	N.a.	N.a.	N.a.
UK	Prynne et al. (83)	1982–1999	1,253	36–53	Diary 5 day
UK	Pot et al. (84)	1982–2011	989	36–64	Diary 3–5 day
UK	Whitton et al. (85)	2000/1–2008/9	1,724 in 2000/1 434 in 2008/9	19–64	Diary 3- or 4-day
UK	Roberts et al. (86)	2008/9–2016/7	1,000	1–100	Diary 4-day

*Hhs, households; FFQ, food frequency questionnaire; N.a., not available; FBS, food balance sheets; HBS, household budget survey*.

Overall, the number of studies conducted in Western European populations was greater, with 45 articles reporting data from 16 Western European countries (Section B of Table 1). For Eastern European countries there were 17 articles reporting data from 10 countries (Section A of Table 1).

Across the different time periods, the last decade (2010–2019) was characterized by a lower number of records as compared to the previous two decades. The evidence was especially scant for Eastern Europe.

### Dietary Data Collection Methodologies

Dietary data methodologies used across studies were predominantly based on national household budget survey data, which was the methodology utilized for 9 out of 10 Eastern European countries and for 12 out of 16 Western European countries. The majority of the publications were based on large national representative samples. Food balance sheets or other routinely collected data from official institutions were used if there were no other sources of information for specific countries and time periods. Most of the included studies provided dietary assessment as population averages with a small number of exceptions including Denmark (50), Germany (59), Greece (61), Italy (67) and two studies from UK (83, 84) which provided individual level assessment of dietary intakes.

### Trends in Dietary Intake Changes

**Figure 2:**
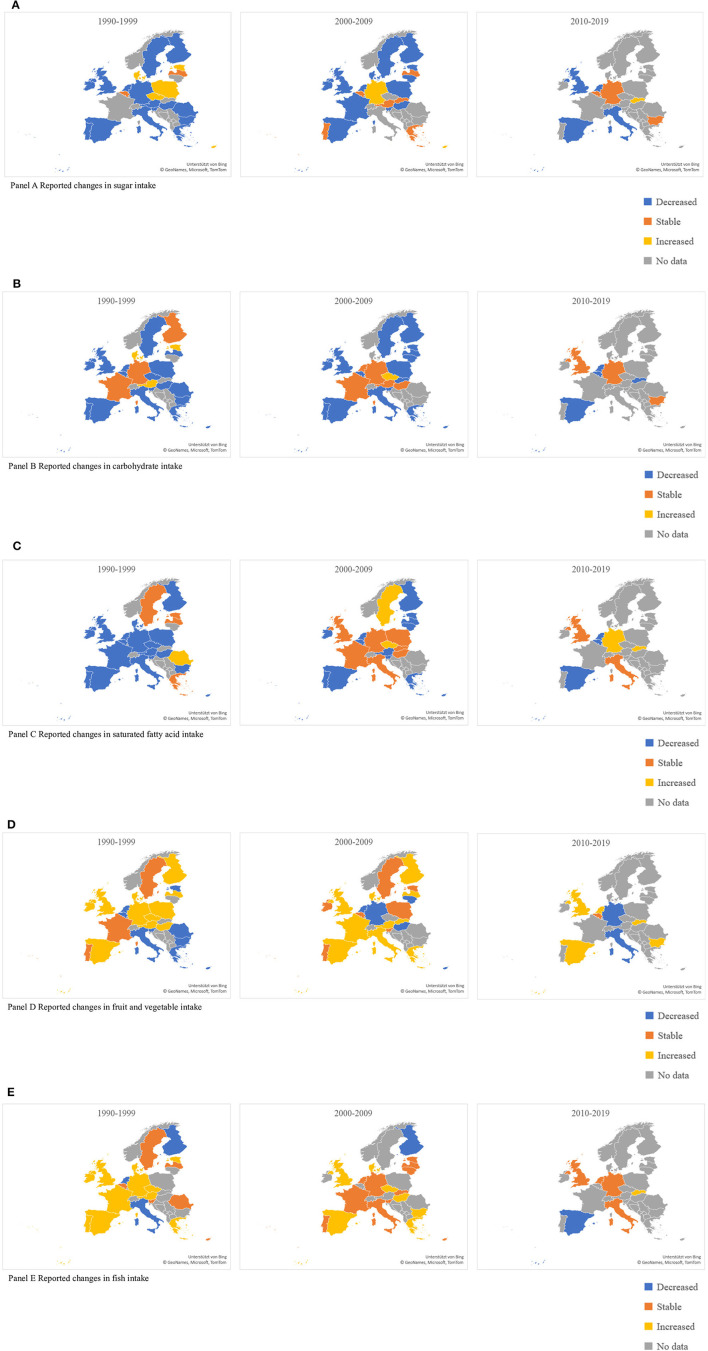
Reported changes in dietary intakes in the European countries (1990–2019), by decade. **(A)** Changes in sugar intake. Decreased, Stable, Increased, No data. **(B)** Changes in carbohydrate intake. Decreased, Stable, Increased, No data. **(C)** Changes in saturated fatty acid intake. Decreased, Stable, Increased, No data. **(D)** Changes in fruit and vegetable intake. Decreased, Stable, Increased, No data. **(E)** Changes in fish intake. Decreased, Stable, Increased, No data.

The majority of studies provided information on selected nutrients, foods and food groups as proxy indicators of the dietary intakes in the studied populations. Among these, we identified saturated fatty acids (SFA), carbohydrates, sugar, fruits and vegetables and fish as the five most commonly assessed dietary indicators with measurements over the studied time periods. Figure 2 provides a summary of the evidence on the changes in consumption trends for these five dietary intake indicators. A more detailed description of results regarding each dietary intake is provided below.

#### Saturated Fatty Acids (SFA)

Overall, a trend of reducing the SFA consumption in the period between 1990 and 2009 could be observed in the majority of Western European countries (Figure 2C). Malta and Greece were exceptions, where the SFA consumption seems to have remained stable in the 90's, followed by a decrease in the next decade. In Sweden and Germany, an increase in SFA was observed in the throughout the whole studied period.

Similar trends with a stable decrease in SFA intakes across all decades could be observed in the majority of the Eastern European countries. Among these, the Czech Republic, Slovenia and Romania (for the period 1990–2000) provided an exception with an overall increase in the SFA consumption over the years.

#### Carbohydrates

The consumption of carbohydrates was also found to follow a decreasing pattern across all decades in the majority of countries, both in Western and Eastern Europe (Figure 2B). The consumption patterns remained stable in Germany and France, whereas an increasing trend was seen in Austria, Denmark and the Czech Republic.

#### Sugar

The consumption of sugar decreased in the majority of the Western European countries (11 out of 16 countries) (Figure 2A). Nevertheless, in some countries, i.e., Cyprus and Malta, there was an increasing trend predominantly in the period between 1990 and 2009.

A decreasing trend of sugar consumption was not so explicit in the Eastern European countries, particularly in the period between 1990 and 2000. However, a tendency of decreasing sugar consumption could be observed for the second decade (2000–2009), except for in Slovakia.

#### Fruits and Vegetables

There was no consistent trend in fruit and vegetable consumption across European countries during the first decade (1990–2000), with half of the countries both in Eastern and Western Europe reporting an increase in intakes, with the remaining half reporting either stable or decreased intakes (Figure 2D). During the second decade (2000–2009), the proportion of countries with reported rising intakes of fruits and vegetables increased. The tendency was stronger in Western European countries (9 out of 16 countries) as compared to Eastern European countries (2 out of 7 countries). Notably, decreases in fruit and vegetable consumption could be observed in some of the Mediterranean countries, i.e., Cyprus and Italy.

#### Fish

There was a trend of increasing fish consumption reported in Western European countries in the period between 1990 and 2000 (Figure 2E). However, the tendency seems to have been retained and even reversed for some countries in the following decades. In particular, countries traditionally characterized by high fish consumption, i.e., Finland and some Mediterranean countries, showed a trend toward decreasing intakes in the last decades. Data on fish consumption available for Eastern European countries revealed a stable consumption pattern throughout the three decades. For the Czech Republic, Slovakia, and Estonia, overall increasing consumption patterns were reported.

### Nutrition Transition Patterns

To allow a comparison of the European transition patterns with global trends to be made, we operationalized the available data on reported dietary intake changes to approximate the nutrition transition patterns according to the pre-defined criteria by Popkin (17). Overall, among the five patterns outlined by the Popkin's nutrition transition model, the European countries could be characterized by experiencing: (a) pattern 4: “the degenerative diseases pattern”, defined as diets high in saturated fat, sugar and refined carbohydrates; and (b) shifts from pattern 4 toward pattern 5: “behavioral change pattern,” defined as diets low in sugar, refined carbohydrates and saturated fat intake and increased fruit, vegetables, and fish intakes.

**Figure 3:**
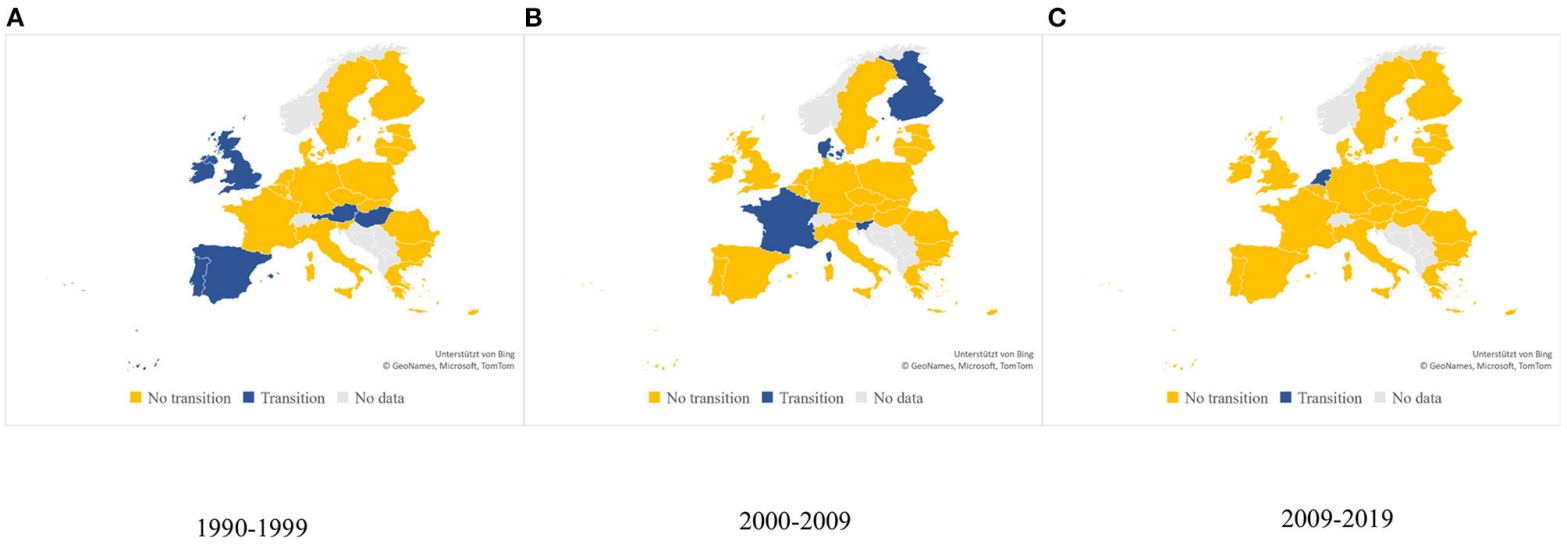
Countries experiencing nutrition transition according to studied decade. **(A)** 1990–1999; **(B)** 2000–2009; **(C)** 2010–2019.

To depict the shifts between pattern 4 to pattern 5, we evaluated whether three or more of the following dietary changes have been observed in each country: (1) reduced sugar intake; (2) reduced refined carbohydrate intake; (3) reduced saturated fat intake; (4) increased fruit and vegetable intake; (5) increased fish intake. Figure 3 provides an overview of the stage of nutrition transition in the European countries according to studied decades. Overall, 12 of the studied 26 European countries could be classified as experiencing a shift from pattern 4 toward pattern 5 of Popkins' nutrition transition model. In Western Europe, 10 of the 16 assessed countries showed a positive dietary change toward pattern 5. The available evidence suggested that the shift had started in the first decade (1990–1999) for 5 of the countries (Austria, Spain, Portugal, Ireland, and the UK). The following decade, four other Western European countries (Denmark, Finland, France and Malta) moved toward pattern 5. In the last decade (2010–2019) one more country, the Netherlands, followed the path. The scarcity of the available data and the lack of a scale do not allow for reporting on the level of the transition (early/advanced) and what proportion of the population is affected by the positive dietary trends. For Sweden, Germany and Belgium, the data indicated stability. For two other countries, Italy and Cyprus, the data indicated “westernization of diet” with a decrease in fruits and vegetable consumption, increase in sugar and lower consumption of fish.

In Eastern Europe, only two countries (Hungary and Slovenia), experienced the positive changes toward pattern 5. For four more countries (Bulgaria, the Czech Republic, Poland, and Latvia) there is some evidence for a beginning transition but not sufficient to be classified as such. For the rest of the countries the data is either lacking or revealing stable, albeit unfavorable trends.

## Discussion

In this narrative review, we provided a summary of published research depicting nutrition transition patterns in Europe over the last three decades (1990–2020) with a focus on comparisons between Eastern and Western European countries. Our literature search resulted in identifying 62 studies eligible for review, among which 17 reported data from Eastern European countries and 45 reported data from Western European countries. To our knowledge, this is the first review that aimed to summarize data on changes in dietary intakes in European populations covering the last three decades characterized by dynamic political, economic and socio-demographic transition between and within European countries. This period has been particularly challenging to Eastern and Central European countries which experienced dramatic societal and political transition after the fall of the Berlin Wall with inevitable consequences to public health.

### Nutrition Transition Toward Healthier Dietary Patterns Across Europe

Our results reveal that there is an overall tendency of increased consumption of healthy foods, i.e., fruits and vegetables and fish, and a decreased consumption of unhealthy nutrients and foods, i.e., SFA and sugar and sugary products. Contrary to the overall transition toward healthier dietary patterns, several Mediterranean countries traditionally characterized by high intakes of fruits and vegetables, showed reversed trends with lowering fruits and vegetable intakes in the recent decades.

Another registered positive trend across most of the European countries was the consistent decline in SFA consumption, starting already before the 1990's (76). It is a main characteristics of the nutrition transition process toward pattern 5 “behavioral change” as defined by Popkin (17). Higher intake of animal and vegetable fats were reported up to the begging of the 90's for the Mediterranean countries, pointed as “westernization of (traditional) diets” (87). However, this last trend typical up to the begging of the 90's reached a peak and was followed by a reverse decrease in SFA consumption in the 90's. The decrease in SFA consumption became also characteristic for some of the Eastern European countries—although for different reasons as compared to the Western countries. The trends for SFA consumption reported here are generally consistent with the estimates by the GBD Study for the same time period (15).

The increases in fruit and vegetables consumption was another positive trend evidenced for half of the European countries. These positive changes are still not overcoming the generally low consumption of fruits and vegetables, for many of the countries. This indicates a need for increasing production and reduction of cross-national availability disparities, through targeted support of countries that lag behind. The estimates of the GBD match closely with our qualitative investigations. The positive trends in fruit and vegetable consumption in Europe have partially been found in the USA where increased consumption of whole fruit, but not of total fruits was reported (88).

The only negative dietary aspect that we describe is the continuous wide scale decrease in complex carbohydrates consumption in all European countries (Western and Eastern) which was discussed also by the GBD study (15). The GBD study depicted specifically the global trend of decreasing whole grain intake occurring also in Europe. This may be partly explained by an overall a decline in the availability of cereals and tubers in Europe until 2011 (e.g., Portugal), as well as by the increasing relative share of meat and dairy products affecting negatively the intake of unrefined and complex carbohydrates. An opposite trend has been observed in the USA where increased consumption of whole grains between 1999–2000 and 2011–2012 has been reported (88).

### Nutrition Transition Differences Between Eastern and Western Europe

The trend toward healthier dietary patterns were obvious in Western European countries since the first studied decade (1990–2000), whereas, in Eastern European countries these pro-health nutrition trends have been emerging only in the two more recent decades (2000–2010 and 2010–2020).

The current evidence suggests that there have been positive changes in diet/nutrition in the Western European countries since the 90's that largely coincided with trends observed in Eastern European countries, albeit showing a tendency to be occurring at a later time period. This phenomenon was especially pronounced for changes in sugar consumption, where most of the Western countries experienced declining trends from the beginning of 1990s, whereas in Eastern European countries sugar consumption started to decline after 2000s. These results were similar to those reported in studies in the USA where sugar consumption started to decline from the middle of the 90's mainly as a result of reduced amount of soft drinks consumption (88).

A positive mild increase in fish consumption has been registered among two thirds of the Western European countries in the 90's. Eastern European countries lagged behind with relatively stable low levels of fish consumption between 1990 and 2010. These results are in line with the results from the GBD study (16). These regional differences in trends have occurred in the context of very different fish consumption levels between countries, well below recommended levels in all European countries. The price of fish, especially in the context of the low price of poultry and meat has been pointed as a major barrier for the low fish consumption and stagnating intake trends (89).

### Inconsistent Dietary Assessment Methodologies and Lack of Systematic Evaluations of Dietary Change

The results from the current review should be interpreted with caution taking into account the variety of methodological approaches for dietary intake assessment used in the studies and the lack of studies systematically evaluating the dietary changes over time across various study populations across Europe. For food and macronutrient intake analysis, we utilized mostly data reported from household budget surveys that exist for many, but not all European countries. Most of the surveys have been standardized using same data collection procedures (45).

### Recommendations for Future Nutrition Transition Research in Europe

There is a lot that can be done on both European and national levels to strengthen the observed positive changes where progress has already been made and also to further stimulate the lagging nations. Efforts and resources need to be directed toward data gathering in a systematic comparable way on individual level through regular representative dietary surveys reported in a standardized / unified way. Wide-scale population level primary prevention can be initiated through both nutrition education, designed to facilitate voluntary adoption of nutrition—related behaviors conducive to health and well-being, and through control of consumers' access to highly processed foods rich in sugar, salt, trans and saturated fats. These preventive actions should include policies toward production and equal access to fruits and vegetables, fish and fish products.

In relation to the nutrition transition process throughout the countries of the European union, the limited available evidence suggests that 12 of the 26 six studied countries are advancing toward the last, pattern five of the nutrition transition theory. The process has started with a more than a decade earlier in Western compared to the Eastern European countries. However, we might question whether these too broadly defined patterns, depicting global tendencies developing over large time spans are of practical importance for the discussion of the European situation. The specific (nutritional) situation in Europe is characterized with a huge diversity of populations and dietary patterns requiring more advanced tools/instruments to allow drawing of a more precise yet standardized evaluation of the nutrition dynamics across and within countries. Recently Popkin and Ng (2021) suggested that it is not inevitable for low and middle income countries experiencing transition toward pattern 4 to reach the same high levels of consumption seen in high-income countries, and the associated negative health impacts (90). Europe urgently needs the development of new dietary assessment metrics and standardized instruments to measure dynamic changes in nutrition transition within and across European populations.

### Strengths and Weaknesses of the Current Review

This narrative review has several strengths. It adds to the evidence from published studies (13–16) and includes additional sources for the decade 2010–2021. It is the first review that provides a general overview on the recent evidence outlining nutrition transition patterns in Europe based on available reports on changes in dietary intakes over the last 30 years. The selected reports are based on nationally representative data, thereby providing an approximation of the diets in the general population. The review methodology followed a consistent, transparent and precise extraction process. Potential limitations should also be considered. These are mostly related to: (1) lack of studies evaluating dietary changes at an individual level; (2) missing information for some European countries and a general under-representation of Eastern European countries; (3) use of different methodologies for dietary assessment; (4) expected reporting bias from generally more health conscience participants (5) qualitative and somewhat fragmented description of the dietary changes based on limited information for small number of nutrients and foods instead of complex quantitative assessment of whole dietary patterns in European populations and (6) insufficient information from specific population groups, i.e., socially disadvantaged, ethnic minority groups. Therefore, the review may not capture the full dimension of the existing nutrition transition across European countries.

## Conclusion

Based on the current review, we observed that European countries seem to have undergone a nutrition transition in the previous three decades, shifting toward healthier dietary behaviors. These processes occurred already in the period 1990–2000 in many Western European, and in the last decades have been also spreading throughout Eastern European countries. Whilst not the predominant outcome, less healthful dietary changes such as moving away from traditional diets i.e., less consumption of fish, were identified in some Nordic and Mediterranean countries. Firm conclusions on trends in dietary changes are hampered by the lack of standardized methodologies depicting changes in dietary intakes over time and the limited coverage of the studied European populations since only those that provided dietary survey data were included in the review. Future studies based on standardized dietary assessment methods and representative for the whole range of populations across Europe are warranted to allow monitoring trends in nutrition transition within and among the European countries.

## Author Contributions

KA conceived the idea of this review paper. KD, RP, NU, TK, SN, GH, and CE conducted the literature search. KD, RP, NU, and GH performed the extraction of the data. KA, KD, and RP wrote the review. All authors provided critical comments, read, and approved the final version of the manuscript.

## Funding

This work was partly supported by The German Federal Ministry of Education and Research (BMBF) (Grant No. 01DS19002).

## Conflict of Interest

The authors declare that the research was conducted in the absence of any commercial or financial relationships that could be construed as a potential conflict of interest.

## Publisher's Note

All claims expressed in this article are solely those of the authors and do not necessarily represent those of their affiliated organizations, or those of the publisher, the editors and the reviewers. Any product that may be evaluated in this article, or claim that may be made by its manufacturer, is not guaranteed or endorsed by the publisher.
